# Redox Potential of Hemoglobin Sub-Micron Particles and Impact of Layer-by-Layer Coating

**DOI:** 10.3390/ijms26157341

**Published:** 2025-07-29

**Authors:** Miroslav Karabaliev, Boyana Paarvanova, Bilyana Tacheva, Gergana Savova, Yu Xiong, Saranya Chaiwaree, Yingmanee Tragoolpua, Hans Bäumler, Radostina Georgieva

**Affiliations:** 1 Department of Medical Physics, Biophysics and Radiology, Medical Faculty, Trakia University, 6000 Stara Zagora, Bulgaria; boyana.parvanova@trakia-uni.bg (B.P.); bilyana.tacheva@trakia-uni.bg (B.T.); gergana.savova@trakia-uni.bg (G.S.); 2 Institute of Transfusion Medicine, Charité-Universitätsmedizin Berlin, 10117 Berlin, Germany; yu.xiong@charite.de (Y.X.); saranya_c@payap.ac.th (S.C.); hans.baeumler@charite.de (H.B.); 3 Department of Pharmaceutical Technology and Biotechnology, Faculty of Pharmacy, Payap University, Chiang Mai 50210, Thailand; 4 Department of Biology, Faculty of Science, Chiang Mai University, Chiang Mai 50200, Thailand; yboony150@gmail.com

**Keywords:** hemoglobin, hemoglobin-based oxygen carriers, redox potential, CCD-technique, layer-by-layer coating

## Abstract

The search for artificial blood substitutes that are suitable for safe transfusion in clinical conditions and in extreme situations has gained increasing interest during recent years. Most of the problems related to donor blood could be overcome with hemoglobin sub-micron particles (HbMPs) that are able to bind and deliver oxygen. On the other hand, the length of the circulation time of HbMPs in the bloodstream strongly depends on their surface properties and can be improved with biopolymer coatings. The redox potential of HbMPs and HbMPs coated with biopolymers using the layer-by-layer technique (LbL-HbMPs) is related to the energy required for electron transfer upon transition from an oxidized to a reduced state. It can be used as a measure of the stability of Hb against oxidation, which is directly connected with its function as an oxygen carrier. The redox potential of Hb, HbMPs, and LbL-HbMPs was determined by a spectroelectrochemical method utilizing the shift of the Soret peak of Hb upon oxidation/reduction of the iron in the heme. The obtained results showed a slight shift in the redox potential of both particle types of about 17 mV towards more negative values compared to the free Hb in the solution. It was demonstrated that the free Hb and the cross-linked Hb in HbMPs and LbL-HbMPs undergo transitions from an oxidized to a reduced state and vice versa several times without Hb destruction. The LbL coating does not affect the redox properties of HbMPs. This ability, as well as the proximity of the obtained redox potentials of Hb, HbMPs, and LbL-HbMPs, indicates that the eventual oxidation of HbMPs in the bloodstream is reversible; thus, HbMPs can be active as artificial oxygen carriers for a longer period of time.

## 1. Introduction

Traditional blood transfusions are essential and life-saving in various treatments, surgeries and emergencies, but face challenges such as the limited availability of safe and compatible donor blood, the risk of transmission of infectious diseases, immunogenic reactions and a short shelf life [1,2,3]. There has been increasing interest in the search for artificial blood substitutes that are suitable for safe transfusion in clinical conditions and in extreme situations in recent decades [4]. Recently, the COVID-19 pandemic led to a significant shortage of blood available for transfusion [5,6,7], again pointing out the importance of the development of safe and effective artificial blood products. The aim is to overcome logistic problems related to the use of donor blood and existing risks of immunomodulation and hemolytic reactions. These limitations require research into a suitable human blood substitute that is readily available, non-toxic and can be administered as needed to all emergency patients independently of their blood groups [8]. The artificial substitutes must be able to perform the functions of hemoglobin (Hb), the main protein in the erythrocytes—also known as red blood cells (RBCs)—providing therapeutic oxygenation, when blood or RBCs are not available for whatever reason.

Human Hb is a quaternary protein composed of four protein subunits (globins). In each protein part of the tetrameric Hb, the Fe^2+^ ion is integrated in the center of a prosthetic group (protoporphyrin IX) by four coordinating bonds with nitrogen atoms. Axially, the Fe^2+^ ion is bound to the protein by the so-called proximal histidine. The oxygen molecule is bound between the iron ions by its sixth coordinating bound and a distal histidine of the protein part. Each Hb molecule can reversibly bind up to four oxygen molecules (O_2_), forming the oxygenated state (oxy-Hb). Then, the O_2_ can be delivered to the tissues and dissociate from Hb due to the low partial oxygen pressure, high CO_2_ concentration and lower pH. The deoxygenated Hb (deoxy-Hb) has a higher affinity to CO_2_ and supports the transport of carbon dioxide from the tissues to the lungs [9]. Hb also plays an important role in the regulation of vascular tonus through its interaction with nitric oxide (NO). When Hb is in its oxygen-free form, it can reversibly bind NO, forming nitrosylated hemoglobin (HbNO). This binding is stable, but NO can be released as needed, which is important for vascular regulation [10,11]. In contrast, the presence of cell-free oxy-Hb in the bloodstream (e.g., in hemolysis) can disrupt the normal vascular function transforming NO to NO_3_ [11]. The stroma-free hemoglobin is unsuitable as a blood substitute due to its short circulation time in vivo, nephrotoxicity, high affinity to NO leading to vasoconstriction, etc. [12,13].

One of the most promising approaches has been to obtain hemoglobin-based oxygen carriers (HBOCs) by utilizing hemoglobin as the oxygen-carrying component [14,15]. Different HBOCs have been already developed and researched. M101 are HBOCs based on hemoglobin obtained from the marine worm Arenicola marina. They show high efficiency as O_2_ carriers with potential anti-inflammatory, antibacterial, and antioxidant properties [16,17,18,19]. Another type of particle (HBOC-201), developed from bovine hemoglobin polymerized with glutaraldehyde, is a suitable blood substitute for the treatment of patients with low hemoglobin (Hb) levels, and can be applied in the treatment of various ischemic and hypoxic diseases. However, HBOC-201 and other similar products show negative effects such as vasoconstriction and increased methemoglobin concentrations [20].

Another promising area of research is linked to hemoglobin nano- and microparticles (Hb-NPs and HbMPs). Hb-NPs, typically ranging from 10 to 200 nm in diameter, aim to increase oxygen-carrying capabilities and simultaneously overcome some of the limitations of traditional blood transfusions [21,22,23,24]. Hb-NPs generally increase oxygen transport due to the increase in total Hb concentration. Their high oxygen affinity prevents the oversupply of oxygen in the tissues and the subsequent vasoconstriction caused by autoregulatory mechanisms. Hb-NPs can also be modified with incorporated antioxidant enzymes such as catalase (CAT) and superoxide dismutase (SOD), enhancing the functional stability of hemoglobin and reducing oxidative stress [25].

The co-precipitation cross-linking dissolution technique (CCD-method), developed in a Charité research group, is a general method used for the production of biopolymer-based microparticles [26]. It can also be utilized to produce hemoglobin microparticles (HbMPs) with controllable sizes determined by the type of inorganic salt used during co-preparation. When CaCO_3_ is used, the resulting particles are in the range of 2–5 µm, whereas the use of MnCO_3_ produces submicron particles with diameters between 0.5 and 1 µm [27,28,29]. The HbMPs prepared by the CCD-method are non-toxic and show a high affinity for oxygen, preventing premature tissue oxygenation and vasoconstriction [30]. In order to improve biocompatibility and protect HbMPs from recognition by immunocompetent cells, their surface has been modified with various biopolymers such as human serum albumin (HSA), hyaluronic acid (HA), and Pluronic acid [27,31].

A basic requirement for all HBOCs is to maintain the iron in the heme in a reduced state (Fe^2+^) to ensure the reversible binding and release of O_2_. There are two main conformations of hemoglobin: a tense T-state, which is more stable in the deoxygenated state, and a relaxed R-state, which is more stable in the oxygenated state. Respectively, the T-state has a low affinity for O_2_, and the R-state has a high affinity for O_2_. When oxygen binds to Fe^2+^, it moves in the plane of the heme group, which causes structural changes that stabilize the R-form. Recently, it was found that the electronic configuration of the heme iron simultaneously changes to a mixed configuration with roughly 57% low-spin Fe^3+^ and 43% low-spin Fe^2+^ [32]. The O_2_ molecule interacts with the iron ion, forming weak σ– but strong π–donor bonds. This is responsible for the overall strong Fe-O_2_ bond required for O_2_ transport. The heterogeneity of the tetrameric structure of hemoglobin also plays a key role in the process contributing to the regulation of O_2_ affinity and cooperativity of O_2_ binding and release. Interactions with O_2_ are reversible as far as the iron in the heme maintains its reduced state. However, there is an alternative reaction leading to the production of superoxide anion radical and methemoglobin (metHb), also known as autoxidation. In metHb, the iron is in an oxidized state (Fe^3+^) and the protein is in a R-like state incapable of binding oxygen [33,34,35,36,37].

The stabilization of the reduced state of the iron in hemoglobin is mainly dependent on the geometry of the heme, which, in turn, is provided by the specific structure of the protein part (the globins) and is influenced by many environmental factors such as pH, ionic strength, ligands, etc. [38,39,40]. Therefore, it is highly important to investigate the redox state of hemoglobin in the HBOCs, taking into account the chemical modifications of the globins in the HBOCs by crosslinking, surface modifications, etc.

The redox potential of hemoglobin is a fundamental property that describes the oxidation capacity of the heme iron, which is sensitive to changes in protein conformation [41]. It is related to the energy required for electron transfer upon transition between the reduced and oxidized states and can be used as a measure of the stability of the protein against oxidation, or to predict how difficult it is to reconstitute it into its reduced state by antioxidants and other cellular reductants such as NADH/NADPH and other enzymes.

One of the most commonly used methods for determining redox potential is the spectroelectrochemical technique. This method facilitates the transition between different redox states of Hb by applying varying electrical potentials to the experimental cell, while the concentration of Hb in a specific redox state is determined through spectrophotometry [42]. The redox potential of heme proteins can be measured directly as they react with electrode surfaces, but the reaction is slow and often irreversible. Due to the complex structure of Hb, the heme-iron center is not accessible for direct electron transfer from the working electrode. This is even more true for HBOCs that capture several Hb molecules in denser or less-dense structures. Therefore, an electron transfer mediator must be used for these reactions. The mediator is reduced or oxidized at the electrode surface and then diffuses to the heme site, where it subsequently reduces or oxidizes the heme-iron center. Upon diffusing back to the electrode surface, the mediator can be re-oxidized or re-reduced [43].

This study aims to answer the question as to whether the redox potential of hemoglobin is altered in HbMPs, as well as in HbMPs, with layer-by-layer (LbL) surface modification by biopolymers (LbL-HbMPs). We applied the spectroelectrochemical technique in order to measure and compare the redox potentials of free Hb, HbMPs, and LbL-HbMPs [44,45]. The LbL coating is based on the deposition of oppositely charged polyelectrolytes. Here, we adsorbed consecutive layers of alginate (Alg), polyethylene mine (PEI), and human serum albumin (HSA) onto HbMPs, creating a polyelectrolyte multilayer film. The LbL deposition of the oppositely charged biopolymers was confirmed by zeta potential and dynamic light scattering (DLS) measurements of the particle size. The final HbMPs and LbL-HbMPs were also observed by transmission electron microscopy (TEM). The redox potential was measured using hexaammineruthenium(III) chloride (Ru(NH_3_)_6_Cl_3_) as the electron transfer mediator.

## 2. Results and Discussion

### 2.1. Fabrication of HbMPs and LbL-HbMPs

**Scheme 1:**
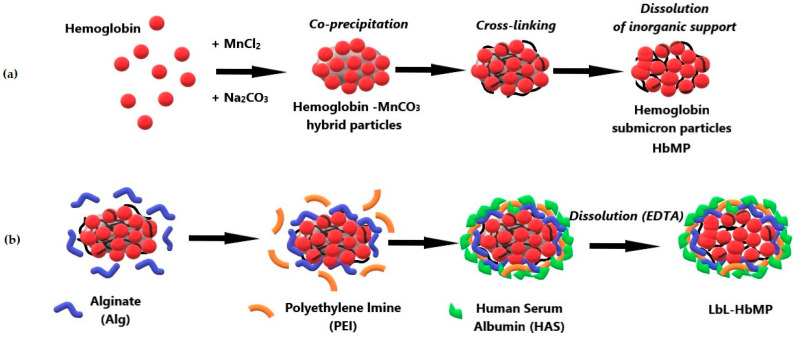
Fabrication procedure of (**a**) hemoglobin microparticles (HbMPs) and (**b**) layer-by-layer-coated hemoglobin particles (LbL-HbMPs).

The HbMPs for this study were fabricated using the CCD-technique with MnCO_3_ as the inorganic template for Hb entrapment (Scheme 1a). This method allows us to obtain stable particles with a high Hb content due to the high affinity of Mn^2+^ to proteins. For our specific preparation protocol, the entrapment efficiency of Hb into the HbMPs varied between 65 and 70% of the initially applied amount of Hb in the fabrication. This result is in a good agreement with previously published data for particles prepared under similar conditions [27,28,29].

The LbL coating was performed by consecutive adsorption of the oppositely charged biopolymers on the HbMPs after the second step (cross-linking) of the CCD-technique before dissolution of the inorganic template (Scheme 1b). Regardless of the fact that the HbMPs are negatively charged, we started with the alginate (Alg), which also carries a negative charge because it appears to enhance and stabilize the negative charge of the HbMPs, preventing their aggregation during further processing.

### 2.2. Characterization of HbMPs and LbL-HbMPs

#### 2.2.1. Electron Microscopy

**Figure 1:**
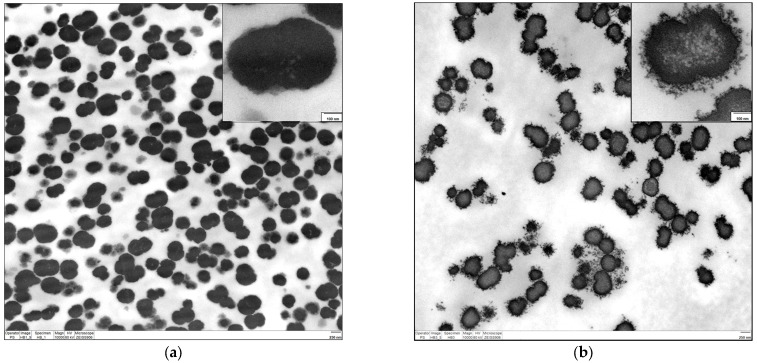
Electron microscopy showing overviews of (**a**) HbMPs and (**b**) LbL-HbMPs. The insets represent enlarged images of the corresponding particles. Scale bars are 250 nm for the overviews and 100 nm for the insets.

Transmission electron microscopy was the method of choice for visualization of the HbMPs’ morphology and to confirm the successful building of the LbL coating. Representative images of the HbMPs and the LbL-MPs are shown in Figure 1a,b. As can be seen, the HbMPs are peanut-like in shape. The LbL coating is well visible as a hairy structure with a thickness of several nanometers on the surface of the particles. The long axis of the majority of HbMPs visible in the cross-section of both images is roughly 700 nm. This is in agreement with previously published measurements of particles prepared by similar protocols [27,28,29,30,31].

#### 2.2.2. Size and Zeta Potential

**Figure 2:**
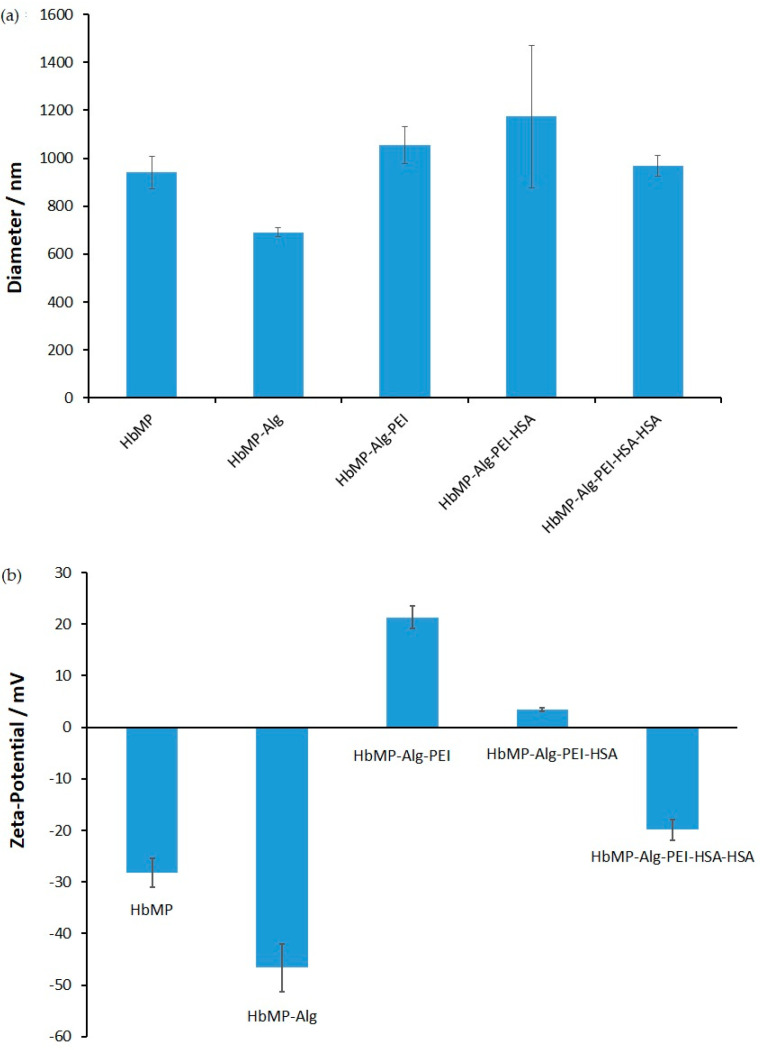
Size (**a**) and zeta potential (**b**) of the HbMPs during the LbL coating procedure. n = 3.

The quality of the HbMPs and the coating process were controlled by measurements of the size and zeta potential. Figure 2 shows the average diameters and the corresponding zeta potentials over three independent preparations and the corresponding LbL coatings.

From the graph in Figure 2a, it can be seen that there is no noteworthy difference between the size of the initial HbMPs (940 ± 67.5 nm) and the final LbL-coated HbMPs (968 ± 42.3 nm). In contrast, the average diameters of the particles measured after each single step of polymer adsorption vary from 691 ± 17.1 nm for HbMP-Alg to 1175 ± 297 nm after adsorption of the third layer (HbMP-Alg-PEI-HSA). The larger diameter and large standard deviation obtained in the measurements of the HbMP-Alg-PEI-HSA sample can be contributed to the formation of more doublet and triplet aggregates, which result in larger diameters calculated from the dynamic light scattering correlation curve.

Interestingly, this suggestion is supported by the zeta potential results of the corresponding samples (Figure 2b). The sample with the highest negative zeta potential (HSA-Alg) has the smallest diameter as measured by dynamic light scattering. It is known that a high zeta potential value is an important prerequisite for stable particle suspension, i.e., the size obtained from dynamic light scattering measurements will be very close to the real particle size, because in a stable suspension, most of the particles are single particles. In contrast, the HbMP-Alg-PEI-HSA sample is almost electro-neutral, with the largest diameter and standard deviation obtained by dynamic light scattering.

### 2.3. Redox Potentials of Hb, HbMPs, and LBL-HbMPs

The reversible oxidation and reduction of free Hb and of Hb cross-linked in microparticles, along with the determination of their redox potentials, was performed by spectroelectrochemistry. Applying different potentials between a working and a reference electrode the oxidation state of Hb changes, which is typically detected by a shift in the Soret peak of the Hb. For metHb, the oxidized Hb, the Soret peak reaches its maximum at 405 nm. In the redox state of Hb, the position of the Soret peak changes depending on the presence and binding of oxygen. Oxygenated Hb (oxyHb) and deoxygenated Hb (deoxyHb) reach their maximum absorption at 413 nm and 430 nm, respectively. For better resolution between the oxidized and reduced state of Hb, we carried out the measurements under an argon atmosphere, which turned Hb into its deoxygenated state. For each potential, the amount of oxidized and reduced Hb is determined by measuring the absorption spectrum of the solution containing either Hb, HbMPs, or LBL-HbMPs. [Ru(NH_3_)_6_]^3+^ and [Ru(NH_3_)_6_]^2+^ ions served as the electron mediators between the working electrode and the iron in the heme of Hb.

**Figure 3:**
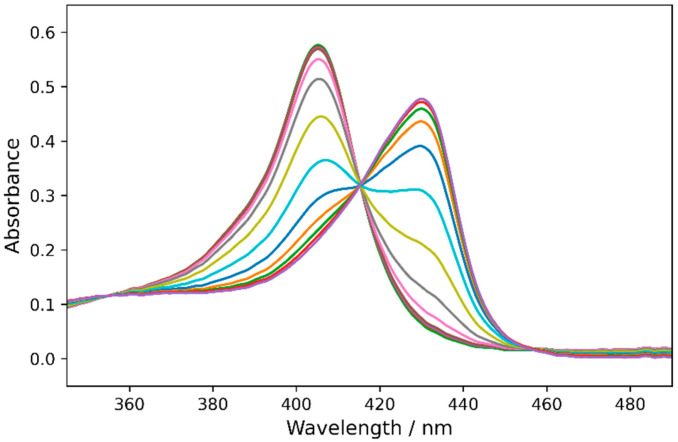
Absorption spectra of Hb at potentials of the working electrode from +400 mV to −300 mV vs. Ag/AgCl reference electrode. The curve with the highest single peak at 405 nm has a potential of +400 mV, and the curve with the highest single peak at 430 nm has a potential −300 mV. Between the pink and the green curve, the potential changes from −25 mV to −175 mV with a 25 mV step.

In Figure 3, the absorption spectra of the hemoglobin at different electric potentials between the working and reference electrodes, starting with +400 mV, are shown.

At an electrode potential of +400 mV vs. Ag/AgCl, all available hemoglobin is oxidized to methemoglobin, and the absorption spectrum has a single maximum peak at 405 nm. By changing the potential to more negative values and approaching the reduction potential, metHb is gradually reduced to deoxyHb. This process is reflected by a gradual absorbance decrease at 405 nm and increase at 430 nm, which are visibly detectable at an electrode potential of −25 mV (the pink curve in Figure 3). The peak at 430 nm reaches its maximum at a potential of about −200 mV as the whole amount of metHb is reduced to deoxy Hb. All individual spectra obtained at different potentials intersect at the so-called isosbestic point. The presence of an isosbestic point indicates the presence of two forms of the investigated substance, which are in different ratios, depending on the applied potential.

**Figure 4:**
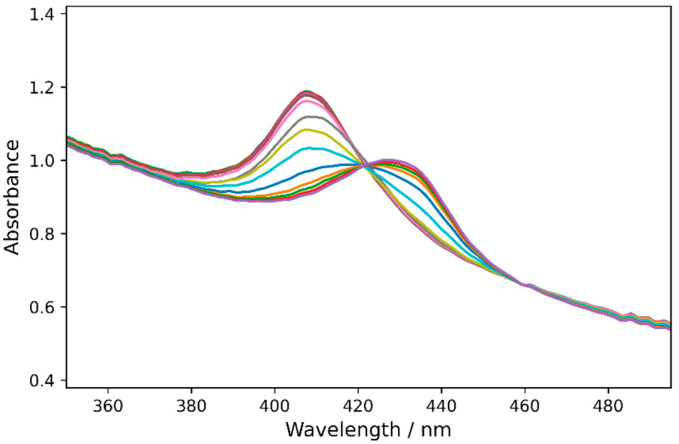
Absorption spectra of HbMPs at potentials of the working electrode from +400 mV to −300 mV vs. Ag/AgCl reference electrode. The curve with the highest single peak at 405 nm has a potential of +400 mV, and the curve with the highest single peak at 430 nm has a potential of −300 mV. Between the pink and the green curve, the potential changes from −50 mV to −250 mV with a 50 mV step.

Similarly, the spectra of HbMP suspensions also show peaks near 405 nm for particles with fully oxidized Hb and at 430 nm for particles with fully reduced Hb (Figure 4). Here, we also observe the gradual reduction of metHb is to deoxyHb during the change in the electrode potential to more negative values and when approaching the reduction potential. The decrease in the metHb peak starts at an electrode potential of +50 mV (the pink curve in Figure 4). The peak at 430 nm reaches its maximum at a potential of about −300 mV as the whole amount of metHb is reduced to deoxy Hb. There is also an isosbestic point in which the individual spectra intersect. In contrast to the spectra of free Hb, an increase in the baseline is observed at all wavelength values due to light scattering by the HbMPs. The scattering from the HbMPs increases at lower wavelengths, which explains the specific shape of the spectra. It should also be noted that the peak at the most positive potential deviates slightly from 405 nm and has its maximum at 407 nm. This could be due to the presence of a small amount of unoxidized Hb molecules inside the HbMPs, possibly due the exchange of electrons between the mediators and the heme iron being sterically hindered.

**Figure 5:**
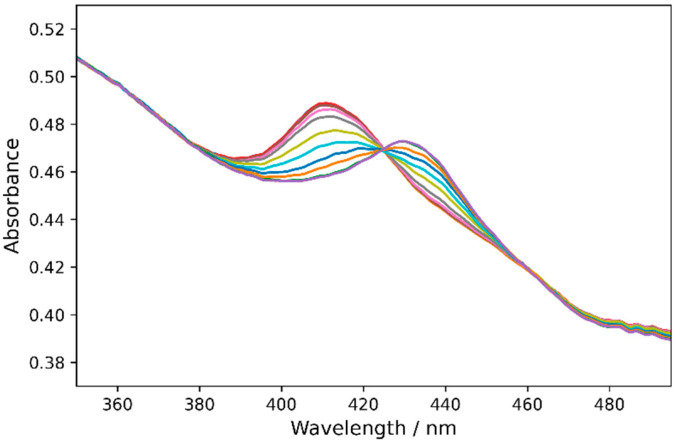
Absorption spectra of LBL-HbMPs at potentials of the working electrode from +400 mV to −300 mV vs. Ag/AgCl reference electrode. The curves with a single peak at 410 nm have potentials of +400 mV, and the curve with the greatest peak at 430 nm has a potential of −300 mV. Between the pink and the orange curve, the potential changes from −50 mV to −200 mV with a 50 mV step.

The absorption spectra of the polyelectrolyte-coated LbL-HbMPs are displayed in Figure 5. It can be seen that the spectra from the oxidized and reduced state of the Hb in these particles are similar to those of the HbMPs. The transition from an oxidized state to a reduced state with the change in potential is clearly visible. The peak of the oxidized state of the Hb in the LbL-HbMPs is slightly more shifted towards higher wavelengths and is located at 410 nm. Therefore, the amount of unoxidized Hb is likely even higher in the polymer-coated particles than in the uncoated HbMPs. The additional layer on the particles may act as an additional shield and barrier to the electron mediators.

The values of the redox potential for Hb in the samples examined are determined by applying a Nernst plot. For a certain value of the applied potential *E*, the ratio between the concentrations of the oxidized form [Ox] and the reduced form [Red] of Hb is given by the Nernst equation as follows:

(1) $$ln\frac{\left[Ox\right]}{\left[Red\right]}=\left(\frac{nF}{RT}\right)E-\;\left(\frac{nF}{RT}\right)E{}^{\circ}$$

where E is the potential applied to the working electrode, E° is the standard equilibrium redox potential, *n* is the number of electrons transferred, *R* and *F* are the gas and Faraday’s constants, and *T* is the absolute temperature. By changing the logarithm from natural to logarithm on base 10, substituting the values of the constants, and assuming the temperature is 20 °C, Equation (1) can be simplified as shown in Equation (2):

(2) $$lg\frac{\left[Ox\right]}{\left[Red\right]}=\frac{n}{0.058}\left(E-E{}^{\circ}\right)$$

The $\frac{\left[Ox\right]}{\left[Red\right]}$ ratio is determined from the experimentally measured absorption spectra. It is assumed that at +400 mV, all Hb present in the solution is oxidized, and the concentration of metHb is maximal, while the concentration of the reduced deoxyHb is zero. In this situation, the peak at 405 nm in the spectrum, measured at +400 mV, is maximal and the absorbance value at this wavelength A_Ox_ corresponds to 100% oxidized Hb ($\left[Ox\right]=100\%$) and 0% reduced Hb ($\left[Red\right]=0\%$). At the other end of the range of applied potentials, −300 mV, all available Hb is reduced, $\left[Ox\right]=0\%$, $\left[Red\right]=100\%$, and the absorbance at 405 nm is minimal. Accordingly, the absorbance value at 405 nm in the spectrum, measured at the potential −300 mV, is defined as $A_{Red}$. At the same wavelength, from each spectrum measured at the given potential E, the absorbance value A_E_ is measured. Thus, the $\frac{\left[Ox\right]}{\left[Red\right]}$ ratio needed to construct the graph of Nernst plot is determined by Equation (3):

(3) $$\frac{\left[Ox\right]}{\left[Red\right]}=\frac{A_{E}-A_{Red}}{A_{Ox}-A_{E}}$$

where the $A_{E}$, $A_{Ox}$, and $A_{Red}$ values are determined at 405 nm from the spectra measured at the respective potentials as described above.

Due to the slight shift in the Soret peak for the metHb of the HbMPs and LbL-HbMPs, the calculations for the Nernst plot of these samples were performed with the absorbance values at 407 nm and 410 nm, respectively.

It can be seen from Equation (2) that the dependence of $lg\frac{\left[Ox\right]}{\left[Red\right]}$ on the applied potential E is linear with a slope equal to n/0.058. The redox potential is determined at the value of $lg\frac{\left[Ox\right]}{\left[Red\right]}=0$, at which E=E°. On the Nernst plot, this is the point where the line crosses the *x*-axis.

**Figure 6:**
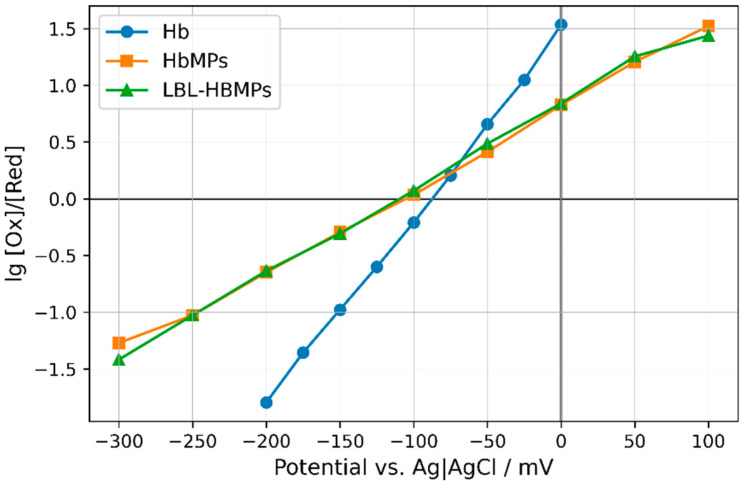
Representative Nernst plots of free hemoglobin (Hb—blue symbols and line) and hemoglobin cross-linked microparticles (HbMPs—red symbols and line; LBL-HbMPs—green symbols and line). The lines represent the linear regressions of the three sets of values.

The Nernst plots obtained for free Hb, HbMPs, and LBL-HbMPs are displayed in Figure 6. In all three cases, the Nernst Equation (1) is satisfied for the given potential ranges, which is reflected in the linear dependency of $lg\frac{\left[Ox\right]}{\left[Red\right]}$ on the applied potential E.

The values of the redox potentials and *n* obtained from the Nernst plots are given in Table 1.

The values of the oxidation-reduction potentials E° are determined by the values of the potentials at which $lg\frac{\left[Ox\right]}{\left[Red\right]}=0$ and the concentrations of the oxidized and reduced form are equal $\;\left[Ox\right]=\left[Red\right]$. As can be seen from Figure 6 and Table 1, the values of E° for the free and cross-linked hemoglobin are very close. For the free hemoglobin, E°= −80.2 mV, for the cross-linked hemoglobin in HbMPs, E° = −96.9 mV, and for the cross-linked hemoglobin in LBL-HbMPs, E° = −97.2 mV. The uncertainties of these values obtained from at least five different experiments for each composition of Hb are similar for Hb and HbMPs and greater for LBL-HbMPs (Table 1). The obtained values of the redox potentials indicate a small negative shift in the redox potential for both HbMPs and LBL-HbMPs. On the other hand, the values of the redox potentials are practically the same in the uncertainty limits, which suggests that the LBL modification of HbMPs does not affect their redox properties.

Furthermore, it is noteworthy that the slope of the Nernst plot of HbMPs and LBL-HbMPs is practically the same, at about 50% lower than that of the free Hb. According to Equation (2), the slope is *n*/0.058, meaning that the measured number of exchanged electrons n is 50% lower in HbMPs and LBL-HbMPs compared to that of the free hemoglobin. The latter may be due to difficult diffusion of the mediator for electron transfer, Ru (NH3)_6_^3+^, to the inner core of the particles, or to the slower electron transfer between the mediator and the cross-linked hemoglobin. At the same time, the very similar values of the slope of the Nernst plots and, respectively, of *n* once again indicate that the LBL modification of HbMPs does not affect their redox properties.

In addition, it has to be mentioned that all samples were measured at least two times in both directions, from full oxidation at +400 mV to full reduction at −300 mV, and vice versa, without any changes in the position and height of the corresponding maximal absorptions. Therefore, the process is fully reversible without any destruction of free Hb in the solution or of the cross-linked Hb in both particle formulations. The slightly more negative redox potential of the Hb in the particles is most probably due to decreased flexibility and changes in the protein conformation caused by the cross-linking.

## 3. Materials and Methods

### 3.1. Materials

Manganese chloride (MnCl_2_), sodium carbonate (Na_2_CO_3_), sodium borohydride (NaBH_4_), ethylene diamine tetra-acetic acid (EDTA), glutaraldehyde (GA), and polyethylenimine (PEI, average M_w_ 750,000 by LS, 50 wt. % in H_2_O) were purchased from Sigma-Aldrich (Taufkirchen, Germany). Phosphate-buffered saline (PBS) was purchased from Fisher Scientific GmbH (Thermofisher, Waltham, MA, USA). Human serum albumin (HSA) solution (Albumnorm 20%) was purchased from Octapharma (Langenfeld, Germany), and alginic acid sodium salt (alginate) from Carl Roth (Karlsruhe, Germany). Sodium chloride 0.9% (NaCl) was purchased from B. Braun (Melsungen, Germany), and dextran (M.W. 70,000 Da) from AppliChem GmbH (Darmstadt, Germany).

Hemoglobin isolated from bovine erythrocytes after hypotonic hemolysis and purified as described in [46] was provided by Biophyll GmbH (Dietersburg, Germany) and stored at −80 °C until use.

### 3.2. Preparation of HbMPs and LbL-HbMPs

#### 3.2.1. CCD-Particle Fabrication Procedure

The hemoglobin microparticles (HbMPs) used in the present study were prepared following the previously described protocol [29] with small modifications. Briefly, the Hb solution was mixed with MnCl_2_ at concentrations of 10 mg/mL and 0.250 M, respectively, and stirred for 5 min at room temperature. Thereafter, an equal volume of 0.25 M Na_2_CO_3_ was rapidly added to start the co-precipitation, and the mixture was stirred for 30 s. The final concentrations during this step were 5 mg/mL Hb, 0.125 M MnCl_2_, and 0.125 M Na_2_CO_3_. After two washing steps with distilled water (centrifugation at 3500× *g* for 2 min Heraeus Biofuge primo R, Thermo Fisher Scientific, Schwerte, Germany), the cross-linking of Hb within the particles was achieved by incubation with glutaraldehyde (final concentration 0.1%) for 1 h. The inorganic MnCO_3_ template was dissolved with 0.25 M EDTA for 30 min, and the imine bonds eventually formed during cross-linking were reduced to stable secondary amines using 0.2 mg/mL sodium borohydride (NaBH_4_). After each of these steps, the particles were washed twice with distilled water. The resulting HbMPs were resuspended in distilled water and stored at 4 °C.

#### 3.2.2. LbL Coating of HbMPs

Layer-by-layer (LbL) self-assembly was used to coat the particles with anionic alginate, cationic PEI, and anionic HSA. The coating was performed on the hybrid particles after Hb cross-linking with glutaraldehyde and before dissolution of the inorganic carbonate template. The particles were resuspended in 0.9% NaCl at a volume concentration of 2% (*v*/*v*), and the polyelectrolytes were consecutively added to the final concentrations of 1.3 mg/mL, 13.3 mg/mL, and 20 mg/mL for alginate, PEI and HSA, respectively. Each layer was adsorbed under stirring for 15 min at room temperature, followed by centrifugation (5000× *g*, 5 min) and two washing steps with distilled water. After the final washing step, the particles were processed as described in the previous chapter (dissolved by EDTA and reduced by NaBH_4_). Finally, the LbL-coated particles were again immersed in 0.9% NaCl containing 20% HSA and stored at 4 °C. The second incubation with HSA after the dissolution of the MnCO_3_ template contributed to the stabilization of the particle suspension with the formation of fewer aggregates.

### 3.3. Hemoglobin Microparticle Characterization

#### 3.3.1. Hemoglobin Content and Entrapment Efficiency

The Hb content of the final HbMPs was determined using a modified alkaline hematin detergent method (AHD-575) [47]. Before measurement, 2% (*v*/*v*) HbMP suspensions was digested by 0.5 mg/mL pronase for 30 min at 45 °C (Roche Diagnostics GmbH, Mannheim, Germany) in order to decrease the scattering of the suspension. Afterwards, the AHD reagent was added at the volume ratio of 1:1, and the absorption of the samples was measured at 575 nm using a microplate reader (PowerWave 340, BioTek Instruments GmbH, Winooski, VT, USA). The entrapment efficiency of Hb was calculated as the percentage of entrapped Hb related to the initial amount applied in the fabrication.

#### 3.3.2. Particle Size and Zeta Potential

Particle size and zeta potential were measured using a Zetasizer Nano series ZS (Malvern Panalytical Ltf, Malvern, UK). Measurements were performed for the final HbMPs as well as after each coating step during the LbL processing. Size measurements were conducted in distilled water applying dynamic light scattering. Zeta potential was determined by measuring electrophoretic mobility using the laser doppler velocimetry technique. Data for the zeta potentials were measured in distilled water at room temperature. Additionally, a light microscope (CKX53, Olympus, Hamburg, Germany) was used to check for the presence of large aggregates.

#### 3.3.3. Transmission Electron Microscopy (TEM)

For transmission electron microscopy, the HbMPs and LbL-HbMPs were dehydrated via incubation in a series of ethanol solutions with gradually increasing solvent concentrations, starting with 10% (*v*/*v*) to absolute ethanol. Subsequently, the ethanol was replaced with a gradually increasing concentration of liquid resin LR-White (Science Services, Munich, Germany) incubated in pure polymer at room temperature overnight. Then, the samples were centrifuged at 1800× *g*, and the supernatant was removed and replaced by fresh LR-White. The samples were transferred into hard gelatin capsules (SERVA Electrophoresis GmbH, Heidelberg, Germany) that were centrifuged at 1800× *g* and polymerized using UV for 48 h at room temperature. For TEM imaging, the specimens were sectioned at 60–70 nm using Ultracut S (Leica Microsystems, Wetzlar, Germany), mounted on mesh grids, and contrasted by 1% phosphotungstic acid and 4% uranyl acetate for 15 min each. The images were obtained with EM 906 (Zeiss, Oberkochen, Germany).

### 3.4. Spectroelectrochemical Measurements

**Figure 7:**
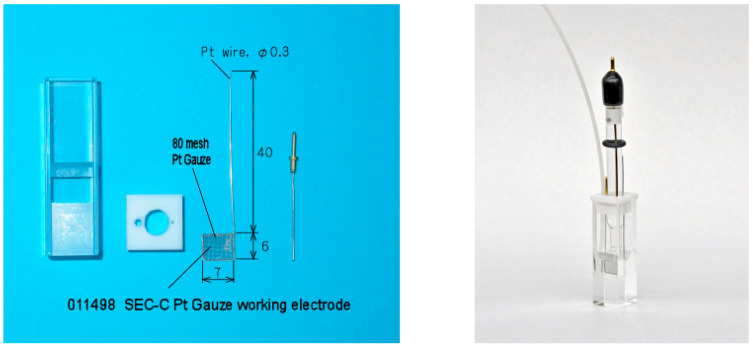
Images of the spectroelectrochemical cell. (**Left**): two-compartment cuvette, Teflon cap, mesh working electrode, and auxiliary platinum (Pt) electrode. (**Right**): Assembled cell with three electrodes and a capillary for the gas supply. (from https://www.als-japan.com/1353.html (accessed on 1 June 2018)).

Determination of the redox potential of Hb and HbMPs was performed in a quartz spectroelectrochemical cell with three electrodes (ALS Co., Ltd., Yokohama, Japan, Figure 7). The two-compartment quartz cuvette with a “wide” compartment (10 × 10 mm) and a “thin layer” compartment (10 × 1 mm) were designed specifically for the spectrophotometric measurements. The redox reactions were carried out on a platinum (Pt) mesh working electrode that was immersed in the thin-layer part of the cuvette. An auxiliary platinum (Pt) electrode was immersed near the working electrode, and a glass pipette with a Ag/AgCl reference electrode was placed over this in the wide compartment. The glass pipette was filled with 3 M NaCl and finished with a salt bridge to provide electrical contact with the sample. The cuvette was tightly sealed by a Teflon cap, through which the three electrodes and a thin capillary for gas supply to the sample were passed. In order to provide an oxygen-free environment, the experiments were carried out under constant argon purging (Ar_2_).

All electrochemical measurements were carried out in a solution of 75 mM KCl with 25 mM phosphate buffer at pH 7. The direct exchange of electrons between the iron in the Hb heme and the working electrode is difficult because of the large distance between them. Therefore, an electron mediator, hexaammineruthenium (III) chloride (Ru(NH_3_)_6_Cl_3_, Strem chemicals), was added to the samples at a concentration of 1 mM.

The mediator is able to exchange electrons with the working electrode, diffuse close to the heme, and easily exchange electrons with the Hb iron in the solution, as well as in the HbMPs, in order to mediate the reduction oxidation reactions (Equations (4) and (5)).

(4) $${\left[Ru{\left(NH_{3}\right)}_{6}\right]}^{3+}+e^{-}\;⇌\;{\left[Ru{\left(NH_{3}\right)}_{6}\right]}^{2+}$$

(5) $${metHb(Fe}^{3+})+{\left[Ru{\left(NH_{3}\right)}_{6}\right]}^{2+}⇌{Hb(Fe}^{2+})+{\left[Ru{\left(NH_{3}\right)}_{6}\right]}^{3+}$$

The electrical potential of the three-electrode system was controlled by a potentiostat/galvanostat (VersaStat 3F, Princeton Applied Research, Oak Ridge, TN, USA); this was varied from −300 mV to +400 mV, and vice versa. Both reactions (5 and 6) were controlled by applying different potentials between the working mesh electrode and the reference electrode. The equilibrium between the corresponding concentrations of the reduced and oxidized form of the Hb was reached within 10 min after applying a given value of the electrode potential. The absorption spectra of the samples were recorded with Cary 60 UV-Vis spectrophotomet (Agilent Technologies, Santa Clara, CA, USA) in the range between 340 and 500 nm. The transition between deoxyHb(Fe^2+^) and metHb(Fe^3+^) is reflected by the shift in the Soret peak of Hb of between 405 and 430 nm.

## 4. Conclusions

Within our study, we demonstrated that the spectroelectrochemical method can be applied to measure the redox potential of HbMP suspensions despite the relatively strong scattering of the particles. The redox potential values of the HbMPs and LbL-HbMPs are slightly more negative than those of the free Hb in the solution. However, coatings using the LbL technique do not affect the redox properties of the HbMPs, as confirmed by their equal redox potentials. Our study has shown the ability of the cross-linked Hb in the HbMPs to undergo transition from an oxidized to a reduced state, and vice versa, by interacting with the electron mediator. This ability, along with the proximity of the obtained redox potentials of Hb, HbMPs, and LbL-HbMPs, indicates that the eventual oxidation of HbMPs in the bloodstream is reversible, and can be managed by intrinsing antioxidants. In a previous investigation, we have shown that the Hb in the HbMPs is stabilized against oxidation [48], and it is assumed that this will also be the case for LbL-HbMPs. Additionally, further developments of the HbMPs with the incorporation of antioxidant enzymes together with Hb will allow HbMPs to remain active as artificial oxygen carriers for a longer period of time in the bloodstream.

## Figures and Tables

**Table 1:** Apparent redox potential and number of electrons transferred for free Hb, HbMPs, and LBL-HbMPs.

Sample	Apparent Redox Potential ^1^ mV	Apparent *n* (Electrons Transferred)
Hb	−80.2 ± 12.8	0.94 ± 0.05
HbMPs	−96.9 ± 14.9	0.49 ± 0.08
LBL-HbMPs	−97.2 ± 21.4	0.53 ± 0.08

^1^ vs. Ag/AgCl.

## Data Availability

The data are available upon request to the authors.
